# A Screening Method for Determining Left Ventricular Systolic Function Based on Spectral Analysis of a Single-Channel Electrocardiogram Using Machine Learning Algorithms

**DOI:** 10.3390/diagnostics16020262

**Published:** 2026-01-14

**Authors:** Natalia Kuznetsova, Aleksandr Suvorov, Daria Gognieva, Zaki Fashafsha, Dmitrii Podgalo, Dinara Mesitskaya, Dmitry Shchekochikhin, Vsevolod Sedov, Petr Chomakhidze, Philippe Kopylov

**Affiliations:** 1 Institute of Personalized Cardiology of The Center “Digital Biodesign and Personalized Healthcare” of Biomedical Science and Technology Park, Federal State Autonomous Educational Institution of Higher Education I.M. Sechenov First Moscow State Medical University of the Ministry of Health of the Russian Federation (Sechenovskiy University), 19991 Moscow, Russia; yourmedstat@gmail.com (A.S.); gognieva_d_g@staff.sechenov.ru (D.G.); fashafshazaki@gmail.com (Z.F.); petr7747@mail.ru (P.C.); fjk@inbox.ru (P.K.); 2 The City Clinical Hospital No. 1 named after N.I. Pirogov, 119049 Moscow, Russia; agishm@list.ru; 3 Department of Cardiology, Functional and Ultrasound Diagnostics of N.V. Sklifosovsky Institute for Clinical Medicine, Sechenov First Moscow State Medical University, 19991 Moscow, Russia; mesitskaya_d_f@staff.sechenov.ru (D.M.); vps52@mail.ru (V.S.); 4 Institute for Clinical Medicine, Sechenov First Moscow State Medical University, 19991 Moscow, Russia; podgalo32@icloud.com

**Keywords:** left ventricular systolic dysfunction, ECG, spectral analysis, machine learning, artificial intelligence

## Abstract

**Background and Objectives:** Given the non-specificity of symptoms and complex methods for diagnosing heart failure, which are not applicable in screening, it is of great importance to develop a simple screening method for identifying systolic dysfunction of the heart based on available biosignals, one of which is a single-channel electrocardiogram (ECG). The method does not require the participation of medical staff. Aim: To create a screening model for detecting left ventricular systolic dysfunction in a complex analysis of single-channel ECG parameters using machine learning algorithms **Methods:** We included 624 patients aged 18 to 90 years. All patients underwent echocardiography and single-channel I-lead ECG recording using a portable electrocardiograph. The left ventricle ejection fraction (LV EF) was determined in the apical 2-chamber and 4-chamber view using the BIPLANE Simpson method and confirmed by two independent experts. Single-channel ECG analysis was performed using advanced signal processing and machine learning techniques. **Results:** For identifying LV EF below 52% in men and below 54% in women, the best result was demonstrated by “Lasso regression”: sensitivity 79.2%, specificity 81.7%, AUC = 0.849. For detection of LVEF below 40%, the “Extra Trees” model was the best, with a sensitivity of 83.1% and a specificity of 82.7%, AUC = 0.972. External testing of the algorithm was conducted on a sample of 600 patients. The accuracy was 98%, specificity 98.4%, and sensitivity 93.5%. **Conclusions:** The results indicate quite high diagnostic accuracy of screening for left ventricular systolic dysfunction when analyzing single-channel ECG parameters using modern signal processing and machine learning technologies.

## 1. Introduction

Left ventricular systolic function (LVSF) is one of the most important indicators of heart function. Left ventricular systolic dysfunction (LVSD), defined as a decrease in the left ventricular ejection fraction to 52% in men and 54% in women or lower, significantly changes patient management [1]. Decreased left ventricular systolic function leads to the development of heart failure (HF), with mildly reduced (41–49%) or reduced ejection fraction (EF).

HF is diagnosed in 1–2% of the adult population in Europe, while in Russia the prevalence is more than 8% of the adult population [2,3]. According to the long-term ESC registry in the outpatient setting, 60% of patients have heart failure with reduced ejection fraction (HFrEF), 24% have heart failure with mildly reduced ejection fraction (HFmrEF), and 16% have heart failure with preserved ejection fraction (HFpEF) [4]. Other reports indicate that the prevalence of HF with mid-range EF is 10–25% [5].

Therefore, the determination of left ventricular ejection fraction (LVEF) is important for all patients, both with cardiac and non-cardiac pathology, especially during surgical treatment, cancer treatment, or other cardiotoxic drugs use. The most common and accessible method for determining LVEF is transthoracic echocardiography (TTE). Echocardiography is operator-dependent and requires an expensive ultrasound machine, which limits its use as a screening method in resource-limited settings. The development of a screening method for determining left ventricular systolic function using machine learning algorithms is a promising direction.

A single-channel ECG is the most accessible biosignal of cardiac function. Unlike a 12-channel ECG, it does not require the presence of medical personnel or special medical equipment. It can be used on inexpensive portable devices everywhere, including wide-scale population screening.

In our previous studies, we determined electrocardiogram (ECG) and pulse wave parameters correlating with the presence of significant left ventricular systolic dysfunction: TA > 33.4 μV with sensitivity 83% and specificity 74%; RonsF > 30.5 Hz—77% and 76%, RoffsF > 28.5 Hz—74% and 63%, accordingly [6]. We have also developed machine learning algorithms on 375 patients to determine systolic and diastolic dysfunction of the left ventricle, demonstrating quite a high diagnostic accuracy (sensitivity and specificity of Lasso regression for LVSD was 92.3% and 90.2% (AUC = 0.922), for LV diastolic dysfunction (DD) 86.2% and 84.5% (AUC 0.878); for the random forest classifier, the sensitivity and specificity for LVSD were 100% and 84.3% (AUC = 0.931), for LV DD 82.8% and 89.3% (AUC 0.854) [6].

However, pulse wave data was used to determine LV systolic function [6]. At the same time, most modern portable devices for recording single-channel ECG do not have a standardized method for recording the pulse wave. This limits the application of our previous algorithms. In this regard, the goal of our study was to identify myocardial systolic dysfunction using single-channel ECG data alone.

## 2. Materials and Methods

**Figure 1:**
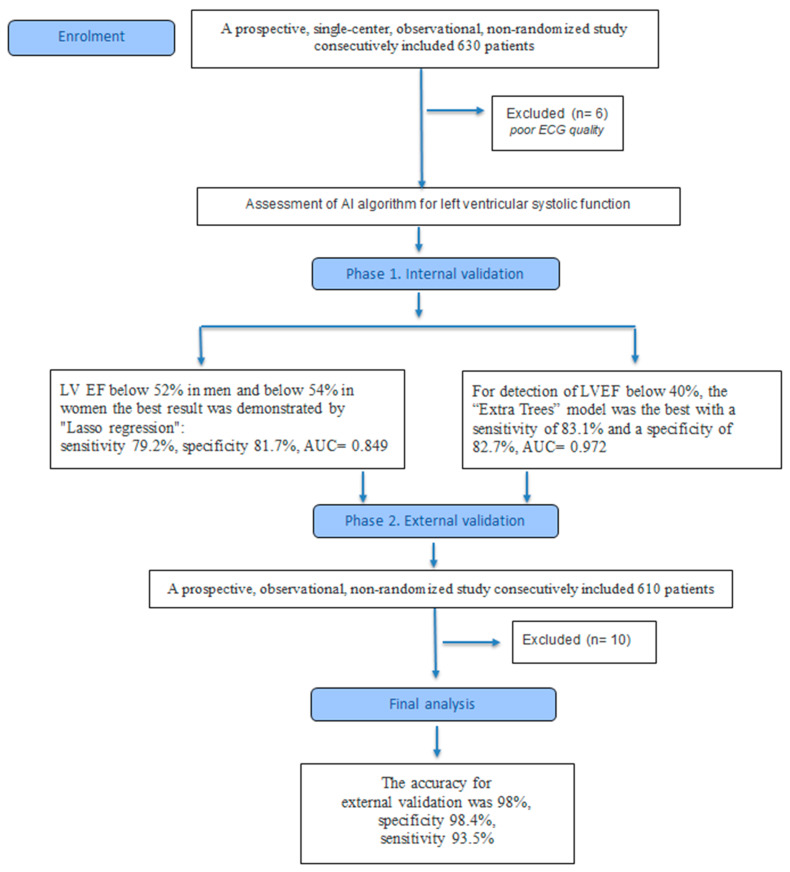
CONSORT 2025 flow diagram. ECG—Electrocardiogram, AI—Artificial intelligence, LV EF—Left ventricular ejection fraction.

A prospective, single-center, observational, non-randomized study consecutively included 630 patients aged 18 years and older. Due to unsatisfactory quality of ECG, 6 patients were excluded. The flow diagram is shown in Figure 1. Patients were treated as outpatients at Clinical Hospital No. 1 of Sechenov University or were hospitalized in the cardiology department of the same hospital.

This protocol was conducted in accordance with the provisions of the Declaration of Helsinki and was approved at the meeting of the local ethics commission of Sechenov University No. 14-19 dated 13 November 2019. The study was registered on the ClinicalTrials.gov website (ID NCT04788342). Each participant signed an informed consent.

In every participant, anamnesis and general examination were collected, transthoracic echocardiography (ECHO) was performed once on a GE VIVID-7 with assessment of left ventricular systolic function in accordance with current recommendations [1], and a single-channel electrocardiogram was recorded once in I standard lead at rest for 1 min using a single-channel portable electrocardiograph “CardioQVARK” (Moscow, Russia). The ECG was performed immediately after ECHO. The ejection fraction was determined in the apical 2-chamber and 4-chamber view using the BIPLANE Simpson method and confirmed by two independent experts.

Registration of a single-channel ECG in I standard lead was performed using a portable electrocardiograph designed in the form of a phone case, with data processed in the original application (registration certificate by the Federal Service for Surveillance in Healthcare of the Russian Federation dated 15 February 2019. N°.RZN 2019/8124).

The clinical characteristics of the patients are presented in Table 1.

More than 200 ECG parameters were obtained, including amplitude, time, frequency, and geometric parameters, according to the slope and angles of attack of different segments of the cardiac cycle. It was planned to obtain a diagnostic accuracy of the algorithm of more than 75%. Statistical calculation of the sample size is more than 520 patients. For the analysis, we used patented machine learning model with parameters of single-channel ECG, most of which were obtained by digital processing of ECG and are not accessible to routine human analysis [6,7]. Some of them are described in Table 2 as an example.

Most of these indicators, which turned out to be significant for identifying systolic dysfunction, are invisible to the human eye, and determine the electrophysiological features of impulse conduction through the myocardium, which depends on the functionality of the heart muscle.

Table 3 shows the ECG parameters that had diagnostic value in identifying myocardial systolic dysfunction.

Next, the diagnostic accuracy indicators for each of the developed models were evaluated. The best result was obtained using the model. The exact set and threshold values of the single-channel ECG parameters are patented by Sechenov University.

### Statistical Analysis

Statistical analysis was performed using Python v3.10 [8].

A total of 624 patients were included in the study. Patients with ejection fraction below 52% in men and below 54% in women were 96 (20.2%), patients with ejection fraction below 40% were 68 (10.9%). Classifier building was performed using PyTorch (version 2.4)-based neural network architectures [9], as well as Lasso and Ridge regression, support vector method, random forest, extra randomized trees, gradient boosting, and an ensemble of classifier data using a soft voting rule [10].

To build and assess the quality of the models, the data were randomly split; thus, internal validation (type 2a from TRIPOD statement) was used [11].

Train/test and validation ratios are presented in Table 4 and Table 5.

**Table 5:** Ejection fraction below 40%.

	Train Set	Validation Set	Test Set
Scikit-learn models and ensemble	436 (22 targets (5%))	-	188 (6 targets (3.2%))
Neural network (PyTorch)	305 (16 targets (5%))	131 (6 targets (4%))	188 (6 targets (3.2%))

Data preprocessing, in the form of feature selection using Lasso regression, boosting algorithm, along with SHAP-value estimation based on the gradient boosting algorithm, was used with standard ML model architectures. Quantitative data were normalized using quantile transformation, and categorical variables were binarized. A 20-fold cross-validation with shuffling was performed. For each method, the best 10 predictors were selected: from Lasso regression on the basis of the absolute values of the coefficients; using the gradient boosting algorithm on the basis of the Gini index; and, in the latter case, on the basis of the value of the obtained SHAP-values. The results of the selection by all 3 methods were combined into a single list of features, which was subsequently used for model building.

A similar data transformation algorithm was used to build neural networks, with the exception of feature selection.

Model training pipeline included similar transformations of quantitative and categorical variables, and hyperparameter selection was performed for each architecture (Lasso regression, support vector machines classifier (SVC), random forest, extra randomized trees, XGBoost), with selection of the best hyperparameters and subsequent calibration during training using isotone regression.

Model training structure: The entire dataset was randomly split (using a pseudorandom number generator with the parameter 9,843,587) into a training set (70% of the total data) and a validation set (the remaining 30% of the total data). The real-valued features of the training set were normalized. The real-valued features of the test set were normalized relative to the training set to eliminate potential biases. First, optimal predictors were selected, followed by a three-fold cross-validation. A separate model was created for each split, and its predictive performance was assessed using the training set. The best model was selected as the final model for use with the test set.

For the training dataset, the best probability threshold was calculated for each architecture (using the Youden index) to obtain sensitivity, specificity, and positive and negative predictive values for the best threshold.

The principle of average predicted probabilities (soft vote) to predict the class labels was used as an ensemble method. For the averaged probabilities, the best threshold was also determined from the training dataset.

## 3. Results

### 3.1. Left Ventricular Ejection Fraction Below Normal

**Figure 2:**
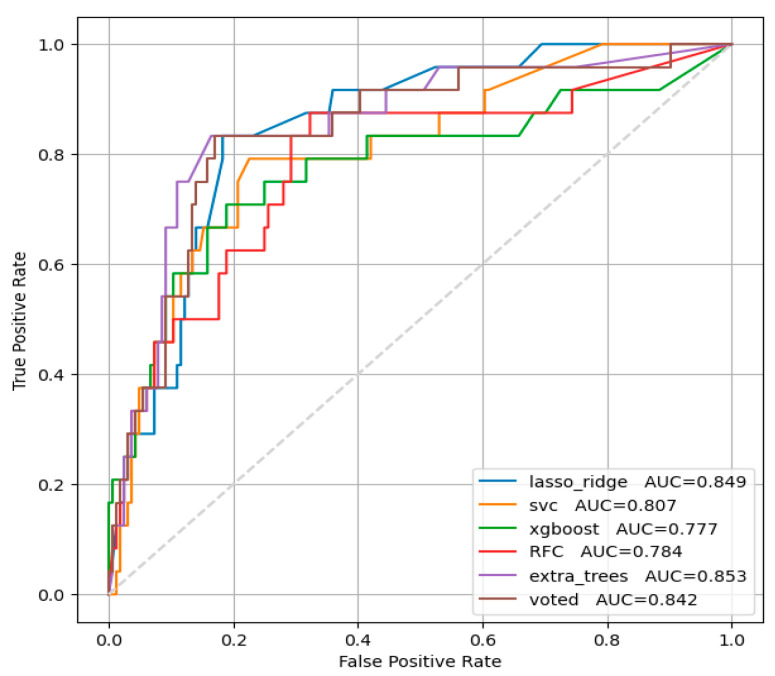
ROC analysis for ejection fraction below normal.

In ejection fraction below 52% in men and below 54% in women, accession selected features were ‘QRSw’, ‘TE1’, ‘Beta’, ‘RA’, ‘PpeakN’, ‘TpTe’, ‘Tons’, ‘RonsF’, ‘TE3’, ‘QRS12energy’, ‘QTc’, ‘Male Gender’, ‘QRSst’, ‘TA’, ‘QRSE2’, ‘QRSE1’, ‘Rpeak’, ‘J80A’, ‘Tpeak’, ‘Speak’, ‘TE2’, and ‘RoffsF’. Figure 2 presents the results for the studied models for reducing systolic function below normal.

The “lasso regression” machine learning model showed the best optimal ratio of sensitivity and specificity for ejection fraction below 52% in men and below 54% in women: 79.2% and 81.7% (AUC = 0.849 [0.767, 0.917]), respectively.

### 3.2. Left Ventricular Ejection Fraction Below 40%

In ejection fraction below 40% in men and women, accession selected features were ‘Pst’, ‘TE3’, ‘SDNN’, ‘PpeakP’, ‘SA’, ‘QRS12energy’, ‘RonsF’, ‘Pfi’, ‘TE4’, ‘Tpenergy’, ‘J80A’, ‘QRSst’, ‘QRSw’, ‘TA’, ‘HFQRS’, ‘Male gender’, ‘Toffs’, and ‘TpTe’.

**Figure 3:**
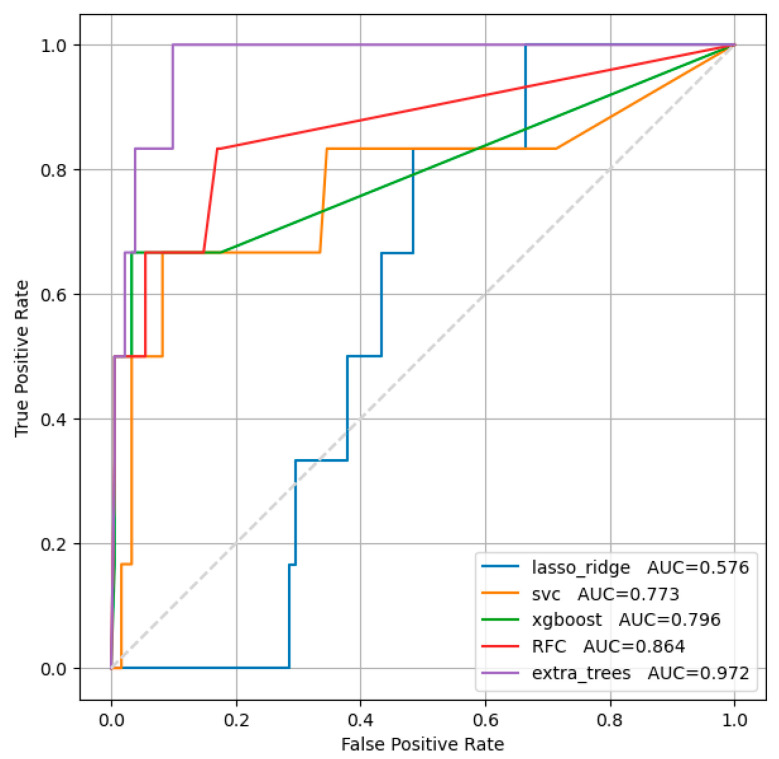
ROC analysis for systolic function below 40%.

Figure 3 presents the results for the studied models for reducing systolic function below 40%.

Among the traditional architectures, the Extra Trees Classifier was the best. Confidence intervals are extremely wide because of the small number of patients with outcome.

The “Extra Trees” machine learning model showed the best optimal ratio of sensitivity and specificity for ejection fraction below 40% in men and in women: 83.1% and 82.7% (AUC = 0.972 [0.927, 0.998]), respectively.

The “Extra Trees” machine learning model included gender, age, smoking, history of diabetes, arterial hypertension, TpTe, VAT, QTc, QT/TQ, HFQRS, JA, J80A, and TA.

The following results were obtained during internal testing of the above-mentioned best models for detection of left ventricular systolic dysfunction using a single-channel ECG. The sensitivity and specificity of the developed method in the detection of a decrease in ejection fraction below the normal value were 78.8% and 80.8%, respectively, and for the detection of a decrease in ejection fraction below 40% were 80.2 and 84.2%, respectively. External validation was planned for next step of our research.

After receiving the results, we conducted external testing of the system. A total of 610 single-channel ECG records were recorded. Due to unsatisfactory quality of ECG, 10 patients were excluded. Then, all patients were examined in clinics in the Tula region. The average age of the examined patients was 59.5 years. There were 63.3% women. Among the patients, 59.3% suffered from arterial hypertension, 2.3% had a history of atrial fibrillation, and 17.5% suffered from diabetes.

A single-channel ECG was recorded using the same procedures as for the training set of patients. The recording was performed immediately before or after echocardiography, in a sitting position after a 5–10 min rest.

After analyzing the ECG using the algorithm we developed, systolic dysfunction was detected in 8.7% of the examined patients (52 people), in 43 of them, a decrease in the systolic function of the left ventricle was confirmed by echocardiography. In addition, in three patients with a decrease in the LV ejection fraction to 48–50%, the algorithm did not show signs of a decrease in systolic function. Thus, among the 600 examined patients, there were 43 true positive results, 9 false positives, 3 false negatives, and 545 true negatives. The accuracy was 98%, specificity 98.4%, sensitivity 93.5%, positive predictive value 82.7%, and negative predictive value 99.5%.

## 4. Discussion

The use of ECG as a screening method for detecting LV systolic dysfunction is a promising approach [12]. Several papers have been published, where a 12-channel ECG processing with artificial intelligence (AI) was used to detect LVEF less than 40% [13,14,15,16,17,18]. Another part of the studies revealed a decrease in LVEF to less than 35% [19,20,21,22].

Katsushika et al. used a dataset of 23,801 ECGs to train a convolutional neural network to identify patients with left ventricular ejection fraction < 40%. When tested on 7196 ECGs, the area under the receiver operating characteristic curve was 0.945 (95% confidence interval: 0.936–0.954). Accuracy was 88.0% ± 3.7%, sensitivity was 74.3% ± 9.0%, and specificity was 89.5% ± 3.9%, respectively. The sensitivity map showed that when LV dysfunction is detected on the ECG, the model focuses on the QRS complex [16]. Similar results with an area under the receiver operating characteristic curve of 0.97, sensitivity of 90%, and specificity of 92% are shown in another study [15]. In intensive care patient, the AI-ECG demonstrates an AUC of 0.83 (95% confidence interval 0.82–0.84) for determining LV systolic disfunction: sensitivity—73%, specificity—78%, negative predictive value—85%, and overall accuracy—76% [14].

Chiou and colleagues showed that using combined signals from leads V5 and V6 of a 12-lead ECG gives the best results for detecting HF [23]. This is a promising message, but it is still necessary to record a 12-lead ECG. The use of a 12-channel ECG requires the involvement of medical personnel, specialized equipment, and space. However, the use of a single-lead ECG to determine LVSD would be more convenient and independent of medical personnel.

In our study, we developed machine learning-based algorithms to detect left ventricular ejection fraction decreases below 52% in men and below 54% in women, as well as LVEF decreases below 40%, using a single-channel ECG, a method that can be used for broad screening, unlike the 12-lead ECG. A separate analysis of the accuracy of our algorithm in patients with a wound causing decreased left ventricular systolic function has not been performed. We have developed a screening method that should be followed by a full evaluation of patients in whom we have identified myocardial dysfunction.

External testing of the algorithm was conducted on a sample of 600 people. Our results with an accuracy of up to 98% in identifying left ventricular systolic dysfunction are quite promising for solving the problem of assessing asymptomatic or low-symptom heart failure, which is vital for patients with heart disease.

According to our data, 99% of the recordings in the overall patient sample, both training and test, were suitable for analysis. It should be noted that the use of a single-lead ECG expands the capabilities of extensive screening without the need for medical equipment but does not allow for a comprehensive analysis of the standard 12-lead ECG.

Undoubtedly, the detection of pathology on a single-lead ECG warrants a comprehensive patient examination.

Despite the positive results obtained, a multicenter pilot study is needed to recommend the use of a standardized method. It is also important to develop an adaptation program for other types of single-channel ECG recorders. Such studies are planned and will begin in the near future.

### The Limits of Our Research

Limitations of our study include the small, single-center test sample. Additional external testing is required. Also noteworthy is the limited number of patients with various etiologies of heart failure. Identifying the cause of decreased systolic function would be important but requires a significantly larger sample size. Currently, this method is not applicable to patients with significant baseline ECG changes, such as complete left bundle branch block, WPW syndrome, or pacemaker rhythm. Furthermore, a limitation of this study is the small number of patients with atrial fibrillation, which may complicate the analysis of some ECG parameters.

## 5. Conclusions

The results of our work indicate promising diagnostic accuracy of screening for left ventricular systolic dysfunction when analyzing single-channel ECG parameters using “Extra tree” machine learning technologies.

## Figures and Tables

**Table 1:** Clinical characteristics, *n* = 624.

Characteristics	Results, *n* (%)
Age	58.0 ± 16.0
Men	261 (41.8%)
Smoking for ≥5 years prior to inclusion	104 (16.7%)
Diabetes mellitus	82 (13.1%)
Hypertension	371 (59.5%)
Stage 1	64 (10.3%)
Stage 2	167 (26.8%)
Stage 3	140 (22.4%)
Ischemic heart disease	151 (24.2%)
Atrial fibrillation at the time of ECG registration	32 (5.1%)
Heart Failure (all classes)	143 (22.9%)
NYHA functional class I	7 (1.1%)
NYHA functional class II	98 (15.7%)
NYHA functional class III	36 (5.8%)
NYHA functional class IV	2 (0.3%)
Aortic insufficiency	
Grade 1	116 (18.6%)
Grade 2	13 (2.1%)
Aortic stenosis	
Grade 1	13 (2.1%)
Grade 2	6 (1.0%)
Grade 3	2 (0.3%)
Mitral insufficiency	
Grade 1	321 (51.4%)
Grade 2	45 (7.2%)
Grade 3	7 (1.1%)
Mitral stenosis	
Grade 1	8 (1.3%)
Grade 2	1 (0.2%)
Left ventricular end diastolic volume	94.6 ± 37.9
LV ejection fraction <52% for men and LV ejection fraction <54% for women	126 (20.2%)
LV ejection fraction <40%	68 (10.1%)
VTI in LV output tract	19.5 ± 4.7
LV DD 1–3 grade	215 (34.5%)
LV DD 2–3 grade	72 (11.5%)

LV—Left ventricular; DD—diastolic dysfunction; NYHA—New York Heart Association; VTI—velocity time integral.

**Table 2:** Description of some digital ECG parameters.

Parameters	Description	Results in the General Group
SBeta	ratio of the maximum modulus of the derivative value at the leading front of the T-wave to the maximum modulus of the value at the trailing front of the T-wave	0.9 ± 0.6
QRSfi	marker of the end of the QRS complex	422.1 ± 135.7
Tfi	T-wave end marker	72.0 ± 241.0
Tpeak	T-wave peak position	394.7 ± 117.7
Tons	point of maximum slope of the T-wave fore front	347.6 ± 114.6
Toffs	point of maximum slope of the T-wave back front	435.6 ± 123.0

**Table 3:** Diagnostic accuracy of ECG parameters in case of decreased systolic function of the left ventricle.

ECG Parameter	AUC	Sensitivity	Specificity
**Left ventricle ejection fraction < 52 for female and < 54% for male**			
TA	0.822	80%	69%
J80A	0.713	77%	66%
RonsF	0.743	81%	77%
RoffsF	0.729	79%	78%
**Left ventricle ejection fraction < 41%**			
TA	0.915	85%	83%
J80A	0.717	76%	73%
RonsF	0.844	82%	82%
RoffsF	0.825	81%	79%

**Table 4:** Ejection fraction below 52% in men and below 54% in women.

	Train Set	Validation Set	Test Set
Scikit-learn models and ensemble	436 (56 targets (12.8%))	-	188 (24 targets (12.8%))
Neural network (PyTorch)	305 (41 targets (13%))	131 (15 targets (11%))	188 (24 targets (12.8%))

## Data Availability

The created database is the patented property of Sechenov University, patent number N° 2021621923 dated 9 September 2021—EDN LVNNZU. In all other cases, please contact the authors.

## Abbreviations

The following abbreviations are used in this manuscript:

AI	Artificial intelligence
DD	Diastolic dysfunction
ECHO	Echocardiography
EF	Ejection fraction
ECG	Electrocardiogram
HF	Heart failure
HFmrEF	Heart failure with mildly reduced ejection fraction
HFpEF	Heart failure with preserved ejection fraction
HFrEF	Heart failure with reduced ejection fraction
LV	Left ventricular
LVEF	Left ventricular ejection fraction
LVSD	Left ventricular systolic dysfunction
LVSF	Left ventricular systolic function
NYHA	New York Heart Association
SVC	Support vector machines classifier
TTE	Transthoracic echocardiography
VTI	Velocity time integral
